# Supplementary material for: Creation and Implementation of Virtual Urogynecology Patient Cases for Medical Student Education
Source: MedEdPORTAL. 2022 May 27;18:11259. doi: 10.15766/mep_2374-8265.11259 (PMC9135914; doi:10.15766/mep_2374-8265.11259)
Supplement: Supplementary file 1 — Case 1 Mixed Urinary Incontinence folderCase 2 Stress Urinary Incontinence folderCase 3 Pelvic Organ Prolapse folderGuide for Virtual Patient Cases.docxGuide for Faculty Debriefing Session.docxSurvey for Virtual Cases.docx [file mep_2374-8265.11259-s001.zip › A. Case 1 Mixed Urinary Incontinence/content/assets/dccfT_kMF7g5vQUT_IVGsMZueg4lhmykw-Urogyn Case 1 Mrs. Green - Visit Summary.pdf]

# Urogyn Case 1: Mrs. Green
# Visit Summary

## Chief Complaint
Leaking urine and peeing all the time

## HPI
Mrs. Green is a 46-year-old Caucasian woman who presents for bladder issues.

She is frustrated because she “pees all the time and leaks urine.”

### Frequency – Day time voiding
Throughout the day, she voids every 1-2 hours

### Frequency – Night time voiding
She gets up 2-3 times a night to urinate

### Sleeping disorders
Denies history of sleep issues

### Description of leakage
She feels strong urges to urinate, but when she gets to the bathroom, usually only voids only a small amount. If she doesn’t make it to the bathroom in time, she leaks.

She also leaks urine when she is exercising, laughing, and coughing.

### Type of leakage that occurs more
She experiences more leakage and is more bothered by leakage when she feels the urge to void and less when she exercises, laughs, and coughs.

## Duration of symptoms
She has had these symptoms off/on for a few years, but in the last year it has worsened.

## Pad use/ Amount leakage/ Amount incontinence
She wears pantyliners all day and changes them about 4x/day because they are wet.

## Impact on quality of life
This has impacted her ability to leave the house, go on vacation, run errands, and work (can't take multiple bathroom breaks as a teacher). She always needs to be near a bathroom. It is also very embarrassing. She's constantly worried that she smells like urine from her leakage.

## Prior evaluations or treatments
She has never taken meds for her symptoms or had prior evaluation with other health care providers for this specific issue.

## Voiding issues
No

## History of urinary tract issues
She has had a couple of urinary tract infections in the past, but these symptoms have resolved with taking antibiotics and she has not required any additional procedures/ work-up. She denies any other urinary tract issues or history.

## Fluid intake
Regarding her diet, she drinks 2 cups of regular coffee in the morning, 1-2 cups of iced tea throughout the day (with stevia), and Diet Coke.

## Bedtime/ Go to sleep
11:00 p.m.

## Restrict fluid intake in evening
No, she drinks up to bedtime

### Other urinary tract/bladder symptoms
She denies other symptoms

### Any vaginal prolapse symptoms/ Feeling vaginal bulge
No

### Any issues with vaginal or perineal splinting to complete defecation
No

## Other Pertinent Questions/ History

### Obstetric history
#### Miscarriage Ectopic pregnancy/ Abnormal pregnancy
None

#### Deliveries
2010: 2 spontaneous vaginal deliveries (SVD) x2), twins at 37+2 weeks of gestation

2013: SVD x 1 at 37+0 weeks of gestation

#### Vaginal tear/ Episiotomy
None

#### Any other obstetric issues
No

### Gynecologic history
#### Menarche
13 yo

#### Contraception
Mirena IUD (placed in 2018)

#### Menstrual history
Amenorrhea since IUD placement

### Last menstrual period (LMP)
2018 (after Mirena IUD placement)

### History of pelvic infections/ Sexually transmitted infection (STI)/ Pelvic inflammatory disease (PID)
No history of sexually transmitted infections

### Sexually active/ intercourse
Yes, monogamous relationship with husband

### Dyspareunia
No issues or pain with sex

### Childbearing status
Has completed childbearing

### Last Pap smear
A few months ago and it was normal

### History of abnormal Pap smears
No

### Any other GYN issues
No

## Past medical history
Seasonal allergies

## Past surgical history
Tonsillectomy (as a child)

Open appendectomy (at 19 years old)

## Medications
Multivitamins, over the counter medication for seasonal allergies as needed

### Medication allergies
No allergies to medication

### Family history
None

### Social history
#### Social history
Married, high school teacher. Exercises 5 days a week.

#### Drink alcohol
1-2 glasses of wine/week

#### Smoke/ Tobacco history
Never smoked. No vaping.

#### Use any other recreational drugs
None

### Review of systems
A review of Mrs. Green’s systems show that all other pertinent systems are negative except as mentioned previously.

## Physical Examination
### Vital signs
#### Heart rate
68

#### Respiratory rate
14

#### Blood pressure
124/76

#### Temperature
98.5 F

### Pain score
0/10

## Additional vital signs
### Height
5 feet 6 inches

### Weight
195 lbs

### Body mass index (BMI)
31.4 $kg/m^2$

## Physical examination parameters
### General
Alert and oriented. No apparent distress (NAD), obese body habitus.

### Head and Neck
Normocephalic, Atraumatic

### Cardiovascular
Regular rate and rhythm (RRR); no rubs, murmurs, or gallops

### Pulmonary
Clear to auscultation bilaterally (CTAB); no wheezes, rhonchi, or rales

### Abdomen
Soft, non tender, non distended. No guarding or rebound. No hepatosplenomegaly.

A well-healed, approximately 10 cm incision in the right lower quadrant. No other surgical incisions/ scars on abdomen.

### Lower extremities
Warm, well perfused bilateral lower extremity. +1 pitting edema from ankle to knee. Palpable peripheral pulses bilaterally.

## Rectal
No masses. No obvious abnormalities.

## Pelvic
### *Parts of pelvic exam*
Speculum exam
Bimanual exam

### *Pelvic exam*
Normal appearing external female genitalia. Normal hair distribution. No clitoral enlargement. No skin changes, rashes, or lesions visualized.

### *Speculum exam*
Normal appearing vagina and cervix with no masses or lesions. No abnormal vaginal discharge. Normal appearing cervix with IUD strings visualized.

### *Bimanual exam*
Approximately 8 week size uterus, anteverted. No adnexal masses palpable. No significant discomfort with examination.

## Urogynecologic
### *Bladder capacity*
350mL

### *Postvoid residual*
40 cc

### *Empty bladder supine cough stress test*
Negative

### *Urethral hypermobility (over 30 degrees)*
Present

### *Prolapse exam*
No vaginal wall laxity on speculum exam

### *Pelvic floor muscle strength*
2/5

## Other Physical Examination Findings/ Office Tests

### Urine dip (Cost: $3 USD)/ Urinalysis (Cost: $45 - $247) USD

| | **Patient Value** | **Normal Values** |
|---|---|---|
| Color | Yellow | Yellow (light/pale to dark/deep amber) |
| Clarity | Clear | Clear or cloudy |
| pH | 7 | 4.5 – 8 |
| Specific gravity | 1.03 | 1.005 – 1.025 |
| Glucose | Negative | $\leq$ 130 mg/d |
| Ketones | Negative | Negative |
| Nitrites | Negative | Negative |
| Leukocyte esterase | Negative | Negative |
| Bilirubin | Negative | Negative |
| Urobilirubin | Negative | Small amount (0.5 – 1 mg/dL) |

*Note: Cost depends on insurance, location of lab, geography.

### Complete blood count (CBC) (Cost: $10 - $200 USD)

Not indicated at this time.

*Note: Cost depends on insurance, location of lab, geography.

### Basic metabolic profile (BMP) (Cost: $10 - $65 USD)

Not indicated at this time

*Note: Cost depends on insurance, location of lab, geography.

## Imaging Studies

### Pelvic ultrasound (Cost: $195 - $700 USD)

Not indicated at this time

*Note: Cost depends on insurance, location of lab, geography.

### Pelvic MRI (Cost: $1,000 - $5,000 USD)

Not indicated at this time

*Note: Cost depends on insurance, location of lab, geography.

### IV pyelogram/ CT urogram (Cost: $1,700 - $10,000 USD)

Not indicated at this time

*Note: Cost depends on insurance, location of lab, geography.

## Office Procedures

### Cystourethroscopy/ Cystoscopy (Cost: $350 - $3,000 USD)

This is not indicated at this time. However, if there was concern for other bladder pathology, such as stone, mass, or the patient had a history of previous bladder surgery (such as a midurethral sling for stress incontinence), it would be recommended. It may also be indicated if the patient fails to respond appropriately to treatment.

*Note: Cost depends on insurance, location of lab, geography.

# Differential diagnosis

- Mixed urinary incontinence
- Overactive bladder
- Nocturia
- Stress urinary incontinence
- Urge urinary incontinence
- Urinary tract infection (UTI)
- Vaginal discharge

## Likely diagnoses

- Mixed urinary incontinence
- Nocturia
- Overactive bladder

## Risk factors for developing symptoms

- Obesity
- Caucasian
- Obstetric history
- Lower extremity edema

## Non-surgical treatment options for symptoms

- Dietary/ lifestyle changes
- Pelvic floor physical therapy with bladder retraining

## Second line treatment

Pharmacotherapy

## Classes of medications for symptoms

- Antimuscarinics (Anticholinergics)
- Beta 3 adrenergic receptor agonist (Mirabegron)

## Clinic/ surgical procedures to be considered for symptoms

- Percutaneous tibial nerve stimulation
- Sacral neuromodulation
- Cystoscopy with OnabotulinumtoxinA injection within the detrusor muscle
