# Supplementary material for: Creation and Implementation of Virtual Urogynecology Patient Cases for Medical Student Education
Source: MedEdPORTAL. 2022 May 27;18:11259. doi: 10.15766/mep_2374-8265.11259 (PMC9135914; doi:10.15766/mep_2374-8265.11259)
Supplement: Supplementary file 1 — Case 1 Mixed Urinary Incontinence folderCase 2 Stress Urinary Incontinence folderCase 3 Pelvic Organ Prolapse folderGuide for Virtual Patient Cases.docxGuide for Faculty Debriefing Session.docxSurvey for Virtual Cases.docx [file mep_2374-8265.11259-s001.zip › B. Case 2 Stress Urinary Incontinence/content/assets/a4A6oxSK3FoREK-P_MSTYeSNW8QsHWMV3-Urogyn Case 2 Mrs. Smith - Visit Summary.pdf]

Urogyn Case 2: Mrs. Smith
Visit Summary

Chief Complaint

Leaking urine with exercise

HPI

Mrs. Smith is a 48-year-old Caucasian woman who presents for bladder issues.

She is frustrated because she has had to stop running and limits exercise due to leakage of urine.

Frequency – Day time voiding

Throughout the day, she voids every 3-4 hours

Frequency – Night time voiding

None

Sleeping disorders

Denies history of sleep issues

Description of leakage

She leaks urine when she is exercising, laughing, and coughing. Leakage occurs both with and without a full bladder.

Duration of symptoms

She has had these symptoms for several years, but in the last year it has worsened. Leakage started after delivery of her second child.

Pad use/ Amount leakage/ Amount incontinence

She wears pantyliners all day and changes them about 3x/day due to leakage.

Impact on quality of life

This has significantly impacted her quality of life, as she has had to modify her lifestyle and limit physical activity. She is a yoga instructor, and this is especially bothersome to her and embarrassing if it occurs when she is teaching yoga.

Prior evaluations or treatments

She has never had prior evaluation with other health care providers for this specific issue. She has tried Kegel exercises on her own without significant improvement.

Voiding issues

None

History or urinary tract issues

When she was in her late teens, she used to get frequent urinary tract infections after intercourse, but this is no longer an issue. She denies other urinary tract issues.

Fluid intake

She drinks mostly water and occasionally tea or soda during the day. She avoids drinking fluids just prior to exercising.

Other urinary tract/bladder symptoms

She denies other symptoms

Any vaginal prolapse symptoms/ Feeling vaginal bulge

None

Any issues with vaginal or perineal splinting to complete defecation

No

Other Pertinent Questions/ History

Obstetric history

Miscarriage Ectopic pregnancy/ Abnormal pregnancy

2005: SAB s/p dilation and curettage

Deliveries

2007: Vacuum assisted vaginal delivery x 1 at 40+1 weeks of gestation

2010: SVD x 1 at 39+0 weeks of gestation

Vaginal tear/ Episiotomy

3rd degree perineal laceration with 1st delivery

How big was largest infant

8 lbs 2 oz

Any other obstetric issues

None

Gynecologic history

Menarche

12 yo

Contraception

History of bilateral tubal ligation

Menstrual history

Regular monthly menses, every 28-30 days, light flow, no intermenstrual bleeding

Last menstrual period (LMP)

15 days ago

History of pelvic infections/ Sexually transmitted infection (STI)/ Pelvic inflammatory disease (PID)

No history of sexually transmitted infections. No active infection.

Sexually active/ intercourse

Yes, monogamous relationship with husband

Dyspareunia

No issues or pain with sex

Childbearing status
Has completed childbearing

Last Pap smear
Las year and it was normal

History of abnormal Pap smears
No

Any other GYN issues
No

Past medical history
GERD

Past surgical history
Bilateral tubal ligation

Medications
Pantoprazole, Multivitamin

Medication allergies
No known drug allergies

Family history
None

Social history
Social history
Married. Masters degree. Yoga instructor. Exercises 5 days a week.

Drink alcohol
1-2 glasses of wine/week

Smoke/ Tobacco history
Never smoked. No vaping.

Use any other recreational drugs
None

Review of systems
A review of Mrs. Smith's systems show that all other pertinent systems are negative except as mentioned previously.

Physical Examination

Vital signs

Heart rate
55

Respiratory rate
13

Blood pressure
120/65

Temperature
98.6 F

Pain score
0/10

Additional vital signs

Height
5 feet 5 inches

Weight
125 lbs

Body mass index (BMI)
20.8 kg/m^2

Physical examination parameters

General

Alert and oriented. No apparent distress (NAD).

Head and Neck

Normocephalic, Atraumatic

Cardiovascular

Regular rate and rhythm (RRR); no rubs, murmurs, or gallops

Pulmonary

Clear to auscultation bilaterally (CTAB); no wheezes, rhonchi, or rales

Abdomen

Soft, non tender, non distended. No guarding or rebound. No hepatosplenomegaly.

Well-healed laparoscopic incision scars. No other surgical incisions/ scars on abdomen.

Lower extremities

Warm, well perfused bilateral lower extremities. No edema bilaterally. Palpable peripheral pulses bilaterally.

Rectal

No masses. No obvious abnormalities.

Pelvic

Parts of pelvic exam

Speculum exam
Bimanual exam

Pelvic exam

Normal appearing external female genitalia. Normal hair distribution. No clitoral enlargement. No skin changes, rashes, or lesions visualized.

Speculum exam

Normal appearing vagina and cervix with no masses or lesions. No abnormal vaginal discharge. Normal appearing cervix without lesions.

Bimanual exam

Approximately 8 week size uterus, anteverted. No adnexal masses palpable. No significant discomfort with examination.

Urogynecologic

Bladder capacity

350mL

Postvoid residual

10 cc

Empty bladder supine cough stress test

Positive

Urethral hypermobility (over 30 degrees)

Present

Prolapse exam

No vaginal wall laxity on speculum exam

Pelvic floor muscle strength

4/5

Other Physical Examination Findings/ Office Tests

Urine dip (Cost: $3 USD)/ Urinalysis (Cost: $45 - $247 USD)

	**Patient Value**	**Normal Values**
Color	Yellow	Yellow (light/pale to dark/deep amber)
Clarity	Clear	Clear or cloudy
pH	7	4.5 – 8
Specific gravity	1.02	1.005 – 1.025
Glucose	Negative	≤ 130 mg/d

Ketones	Negative	Negative
Nitrites	Negative	Negative
Leukocyte esterase	Negative	Negative
Bilirubin	Negative	Negative
Urobilirubin	Negative	Small amount (0.5 – 1 mg/dL)

*Note: Cost depends on insurance, location of lab, geography.

Complete blood count (CBC) (Cost: $10 - $200 USD)

Not indicated at this time.

*Note: Cost depends on insurance, location of lab, geography.

Basic metabolic profile (BMP) (Cost: $10 - $65 USD)

Not indicated at this time

*Note: Cost depends on insurance, location of lab, geography.

Imaging Studies

Pelvic ultrasound (Cost: $195 - $700 USD)

Not indicated at this time

*Note: Cost depends on insurance, location of lab, geography.

Pelvic MRI (Cost: $1,000 - $5,000 USD)

Not indicated at this time

*Note: Cost depends on insurance, location of lab, geography.

IV pyelogram/ CT urogram (Cost: $1,700 - $10,000 USD)

Not indicated at this time

*Note: Cost depends on insurance, location of lab, geography.

Office Procedures

Cystourethroscopy/ Cystoscopy (Cost: $350 - $3,000 USD)

This is not indicated at this time. However, it could be considered if she had a history or prior surgical treatment for stress urinary incontinence, recurrent urinary tract infections (URIs), stones, or hematuria.

*Note: Cost depends on insurance, location of lab, geography.

Urodynamic testing (Cost: $350 - $1,000 USD)

This is not indicated at this time. Urodynamic testing could be considered if the patient had symptoms that were not consistent with physical exam findings, prior history of incontinence procedures, or neurologic disorders that could complicate their incontinence history.

Differential Diagnosis

- Mixed urinary incontinence
- Stress urinary incontinence
- Urge urinary incontinence
- Overflow urinary incontinence
- Urinary tract infection (UTI)
- Vaginal discharge

Likely diagnosis

Stress urinary incontinence

Risk factors for developing symptoms

- Caucasian
- Female gender
- Obstetric history
- Age

Non-surgical treatment options for symptoms

- Pelvic floor physical therapy
- Incontinence pessary
- Over the counter incontinence device (e.g., Poise Impressa [Kimberly-Clark; Irving, TX])

Clinic/ surgical procedures to be considered for symptoms

- Midurethral sling (transobturator or retropubic approach)
- Urethral bulking
- Mini-sling
- Autologous fascial sling
- Burch colposuspension
