# Supplementary material for: Creation and Implementation of Virtual Urogynecology Patient Cases for Medical Student Education
Source: MedEdPORTAL. 2022 May 27;18:11259. doi: 10.15766/mep_2374-8265.11259 (PMC9135914; doi:10.15766/mep_2374-8265.11259)
Supplement: Supplementary file 1 — Case 1 Mixed Urinary Incontinence folderCase 2 Stress Urinary Incontinence folderCase 3 Pelvic Organ Prolapse folderGuide for Virtual Patient Cases.docxGuide for Faculty Debriefing Session.docxSurvey for Virtual Cases.docx [file mep_2374-8265.11259-s001.zip › C. Case 3 Pelvic Organ Prolapse/content/assets/I28hfM559hWCDxd4_PNobn6_HiIXcup5l-Urogyn Case 3 Mrs. Jones - Visit Summary.pdf]

Urogyn Case 3: Mrs. Jones
Visit Summary

Chief Complaint
Something's falling out down there

HPI
Mrs. Jones is a 65 year-old Caucasian woman who present to the gynecology clinic for a "something falling out" from her vagina.

Other associated symptoms
She reports symptoms of vaginal bulge and constant pressure.

Other associated symptoms, specifically, vaginal splinting
Throughout the day, she voids every 3-4 hours.

Frequency
She feels bulge symptoms daily, worse towards the end of day, especially if she is more active.

Duration
She has had these symptoms off/on for approximately 4-5 years, but in the last few months it has gotten worse and more noticeable.

Prior evaluations or treatments
She has previously seen her general gynecologist for this issue and was only given reassurance because her symptoms were not bothersome at that time. She has not tried any prior treatments or undergone other evaluation for this issue.

Impact on quality of life
This has impacted her ability to enjoy her normal activities, like gardening and walking, as the bulge is very bothersome to her with these activities.

Impact on intercourse

She continues to be sexually active without issues.

Voiding issues

Sometimes she feels like she needs to push on the bulge in order to empty her bladder completely. Otherwise, no issues with voiding.

Lower urinary tract issues

Apart from having to push the bulge at times in order to complete urination, she denies any other urinary tract issues or history.

Issues with bowel movements

Occasional constipation, managed with diet and Miralax as needed. Denies fecal incontinence.

Other Pertinent Questions/ History

Obstetric history

Miscarriage Ectopic pregnancy/ Abnormal pregnancy

Spontaneous abortion x 1

Deliveries

1978: Forceps assisted vaginal delivery x 1 at 40+2 weeks of gestation

1980: SVD x 1 at 40+0 weeks of gestation

1982: SVD x 1 at 38+3 weeks of gestation

1985: Cesarean section x 1 for breech presentation at 39 weeks gestation

Vaginal tear/ Episiotomy

History of episiotomy x 2

How big was largest infant

8 lbs 7 oz

Any other obstetric issues
None

Gynecologic history

Menarche
13 yo

Contraception
N/A

Menstrual history
Postmenopausal

Last menstrual period (LMP)
Age 51

History of pelvic infections/ Sexually transmitted infection (STI)/ Pelvic inflammatory disease (PID)
No history of sexually transmitted infections. No active infection.

Sexually active/ intercourse
Yes, monogamous relationship with husband. She avoids sex due to embarrassment with prolapse, but desires to maintain sexual function.

Dyspareunia
No issues or pain with sex.

Last Pap smear
A few months ago and it was normal.

History of abnormal Pap smears
No

Postmenopausal bleeding
No

Symptoms of vaginal dryness to atrophy
No, but uses lubrication for intercourse.

Any other GYN issues
No

Past medical history
Obstructive sleep apnea, (OSA), type 2 diabetes, hypertension, obesity, COPD. She states that these conditions are well managed and last saw her primary care doctor one month ago.

Past surgical history
Cesarean section x1, dilation and curettage (for spontaneous abortion)

Medications
Metformin, Hydrochlorothiazide, Aspirin, Albuterol, Miralax

Medication allergies
No allergies to medication.

Family history
Mother: History of hysterectomy for prolapse, hypertension

Sister: History of hysterectomy for prolapse

Social history
Social history
Married, retired teacher. Walks 2-3 miles, 5-6 days a week.

Drink alcohol
1-2 glasses of wine/week.

Smoke/ Tobacco history
Former smoker, approximately 1/2 pack per day x 20 years. Quit 15 years ago.

Use any other recreational drugs
None.

Review of systems
A review of Mrs. Jones' systems show that all other pertinent systems are negative except as mentioned previously.

Physical Examination

Vital signs

Heart rate

70

Respiratory rate

13

Blood pressure

130/80

Temperature

98.5 F

Pain score

0/10

Additional vital signs

Height

5 feet 5 inches

Weight

200 lbs

Body mass index (BMI)

33.3 kg/m^2

Physical examination parameters

General

Alert and oriented. No apparent distress (NAD), obese body habitus.

Head and Neck

Normocephalic, Atraumatic

Cardiovascular

Regular rate and rhythm (RRR); no rubs, murmurs, or gallops

Pulmonary

Clear to auscultation bilaterally (CTAB); no wheezes, rhonchi, or rales

Abdomen

Soft, non tender, non distended. No guarding or rebound. No hepatosplenomegaly.

A well-healed low transverse incision. No other surgical incisions/ scars on abdomen.

Lower extremities

Warm, well perfused bilateral lower extremities. No edema bilaterally. Palpable peripheral pulses bilaterally.

Rectal

No masses. No obvious abnormalities.

Pelvic

Parts of pelvic exam

Speculum exam
Bimanual exam

Pelvic exam

Normal appearing external female genitalia. Normal hair distribution. No clitoral enlargement. No skin changes, rashes, or lesions visualized.

Speculum exam

Normal appearing vagina and cervix with no masses or lesions. Increased pallor and loss of rogation consistent with vaginal atrophy. No abnormal vaginal discharge. Normal appearing cervix without lesions.

Bimanual exam

Small, anteverted uterus. No adnexal masses palpable. No significant discomfort with examination.

Urogynecologic

Postvoid residual

15 cc

Urethral hypermobility (over 30 degrees)
Present

Pelvic floor muscle strength
2/5

Prolapse exam (POPQ)
Aa (anterior wall): 0
Ba (anterior wall): 0
C (cervix or cuff): -3
Gh (genital hiatus): 6
PB (perineal body): 3 (not seen)
TVL (total vaginal length): 9 (not seen)
Ap (posterior wall): -2 (not seen)
Bp (posterior wall): -2 (not seen)
D (posterior fornix): -5 (not seen)

Other Physical Examination Findings/ Office Tests

Urine dip (Cost: $3 USD)/ Urinalysis (Cost: $45 - $247 USD)

Not indicated at this time.

*Note: Cost depends on insurance, location of lab, geography.

Complete blood count (CBC) (Cost: $10 - $200 USD)

Not indicated at this time.

*Note: Cost depends on insurance, location of lab, geography.

Basic metabolic profile (BMP) (Cost: $10 - $65 USD)

Not indicated at this time

*Note: Cost depends on insurance, location of lab, geography.

Imaging Studies

Pelvic ultrasound (Cost: $195 - $700 USD)

Not indicated at this time

*Note: Cost depends on insurance, location of lab, geography.

Pelvic MRI (Cost: $1,000 - $5,000 USD)

Not indicated at this time

*Note: Cost depends on insurance, location of lab, geography.

Office Procedures

Multichannel urodynamic testing (Cost: $350 - $1,000 USD)

This is not indicated at this time, but may be indicated in the future depending on treatment plan.

*Note: Cost depends on insurance, location of lab, geography.

Office simple cystomrty (Cost: $79 - $190 USD)

This is not indicated at this time, but may be indicated in the future depending on treatment plan.

*Note: Cost depends on insurance, location of lab, geography.

Differential Diagnosis

- Anterior pelvic organ prolapse
- Apical pelvic organ prolapse/ Uterine prolapse
- Posterior pelvic organ prolapse
- Vaginal mass
- Periurethral mass

Likely diagnoses

- Anterior pelvic organ prolapse
- Apical pelvic organ prolapse/ Uterine prolapse

Risk factors for developing symptoms

- Obesity
- Caucasian
- Age
- Obstetric history
- Constipation
- COPD

- Family history of prolapse

Non-surgical treatment options for symptoms

- Pelvic floor physical therapy
- Pessary

Surgical procedures to be considered for symptoms

- **Vaginal pelvic reconstructive surgery with or without hysterectomy:** Anterior vaginal wall repair, vaginal hysterectomy, uterosacral suspension or sacrospinous suspension; anterior repair, sacrospinous hysteropexy (without hysterectomy)

- **Abdominal (can be done via laparotomy laparoscopically or robotically) sacropexy, abdominal hysterectomy; abdominal sacrohysteropexy (without hysterectomy)**

- **Vaginal obliterative surgery with or without hysterectomy:** Complete colpoclesis, vaginal hysterectomy; LeFort colpoclesis (without hysterectomy)

Other procedures to be considered if considering surgery

The patient, Mrs. Jones, may be at risk for occult stress incontinence (stress urinary incontinence after prolapse reduction). Office evaluation that can be performed to assess for this condition prior to surgery include office cystometry or multichannel urodynamic testing.

If the patient tests positive for stress incontinence, surgery for stress incontinence may be performed at the time of prolapse surgery.
