# Supplementary material for: Creation and Implementation of Virtual Urogynecology Patient Cases for Medical Student Education
Source: MedEdPORTAL. 2022 May 27;18:11259. doi: 10.15766/mep_2374-8265.11259 (PMC9135914; doi:10.15766/mep_2374-8265.11259)
Supplement: Supplementary file 1 — Case 1 Mixed Urinary Incontinence folderCase 2 Stress Urinary Incontinence folderCase 3 Pelvic Organ Prolapse folderGuide for Virtual Patient Cases.docxGuide for Faculty Debriefing Session.docxSurvey for Virtual Cases.docx [file mep_2374-8265.11259-s001.zip › D. Guide for Virtual Patient Cases.docx]

**Medical Student Virtual Urogynecology Patient Cases**

**Guide**

**Instructions**

To maximize your learning in reviewing the virtual patient cases, we recommend

1. Reading the background materials (listed below) in advance of completing the online virtual patient cases.
2. Arrange time with your gynecology/ urogynecology faculty or fellow to discuss and debrief on these cases.

**Background readings/ materials**

1. Pelvic Organ Prolapse (ACOG Bulletin #214, November 2019)
2. Urinary Incontinence in Women (ACOG Bulletin #155, November 2015)
3. Evaluation of Uncomplicated Stress Urinary Incontinence in Women Before Surgical Treatment (ACOG Committee Opinion #603, June 2014)
