# Supplementary material for: Creation and Implementation of Virtual Urogynecology Patient Cases for Medical Student Education
Source: MedEdPORTAL. 2022 May 27;18:11259. doi: 10.15766/mep_2374-8265.11259 (PMC9135914; doi:10.15766/mep_2374-8265.11259)
Supplement: Supplementary file 1 — Case 1 Mixed Urinary Incontinence folderCase 2 Stress Urinary Incontinence folderCase 3 Pelvic Organ Prolapse folderGuide for Virtual Patient Cases.docxGuide for Faculty Debriefing Session.docxSurvey for Virtual Cases.docx [file mep_2374-8265.11259-s001.zip › E. Guide for Faculty Debriefing Session.docx]

**Faculty Debrief Guide**

**Goals:**

- Practice oral presentation skills: Have the learner present at least 1 virtual patient case and provide feedback (as if the learner evaluated the virtual patient case in clinic and are now presenting the patient to you)
- Review and discuss the learners’ reaction to the virtual patient cases
- Assist in solidifying the learning objectives from each virtual patient case
- Review the “take-home messages” of the virtual patient cases
- Address any questions that the learner may have

**Debriefing Questions:**

1. How did it feel going through the virtual patient cases?
2. What is something you specifically learned about pelvic floor disorders from this case?
3. What are at least 2 things you learned from this virtual patient case that you can apply to your future patient encounters?
4. What questions do you have about the virtual patient case?
5. Is there anything else you would like to discuss?
