# Supplementary material for: Creation and Implementation of Virtual Urogynecology Patient Cases for Medical Student Education
Source: MedEdPORTAL. 2022 May 27;18:11259. doi: 10.15766/mep_2374-8265.11259 (PMC9135914; doi:10.15766/mep_2374-8265.11259)
Supplement: Supplementary file 1 — Case 1 Mixed Urinary Incontinence folderCase 2 Stress Urinary Incontinence folderCase 3 Pelvic Organ Prolapse folderGuide for Virtual Patient Cases.docxGuide for Faculty Debriefing Session.docxSurvey for Virtual Cases.docx [file mep_2374-8265.11259-s001.zip › Educational_Summary_Report_11259.pdf]

MedEdPORTAL® The AAMC Journal of Teaching and Learning Resources

Original Publication Open Access

Creation and Implementation of Virtual Urogynecology Patient Cases for Medical Student Education

Jacqueline Y. Kikuchi, MD*, Margot Le Neveu, MD, Shannon Arnold, MSc, Austin Offnick, Keila S. Muñiz, MD, Prerna Pandya, MD, Rehan Feroz, DO, Jaime B. Long, MD, Lindsay R. Ledebur, MA, Danielle Patterson, MD, Chi Chiung Grace Chen, MD, MHS

*Corresponding author: jacqueline.kikuchi@gmail.com

Abstract

Introduction: Urogynecologic disorders are highly prevalent, and many physicians across various specialties will encounter and care for patients with pelvic floor disorders. Yet most medical students have had limited to no experience in diagnosing and managing pelvic floor disorders, resulting in a gap in clinical education. **Methods:** Three virtual and interactive urogynecologic patient cases were developed on an e-learning platform with an overall goal of increasing clinical exposure to various pelvic floor disorders. The cases were integrated into the medical student obstetrics and gynecology clerkship during the 2020-2021 academic year ($n = 40$). Participants provided feedback regarding usability, acceptability, and educational value of the cases. **Results:** Twenty-one students (52%) completed the survey. Ninety percent ($n = 19$) agreed or strongly agreed that they were satisfied with the cases, and 71% ($n = 15$) agreed or strongly agreed that they would recommend the virtual patient cases to other students. All students ($n = 21$) felt that the format was easy to use and reported that the cases were appropriate for their level of learning. Most students felt that the cases increased or significantly increased their confidence regarding nonsurgical and surgical management options for pelvic floor disorders. **Discussion:** Our findings suggest that these interactive virtual patient cases are an acceptable, valuable, and effective tool for learners. Utilizing the cases can help mitigate existing disparities in exposure to pelvic floor disorders both highlighted by and preceding the COVID-19 pandemic.

Keywords
Pelvic Floor Disorders, Pelvic Organ Prolapse, Urinary Incontinence, OB/GYN, Online/Distance Learning, Urology, Virtual Learning

Educational Objectives

By the end of this activity, learners will be able to:

1. Conduct a thorough patient interview and obtain an appropriate history for the following pelvic floor disorders: pelvic organ prolapse, stress urinary incontinence, and mixed urinary incontinence.
2. Describe the components of a gynecologic and urogynecologic physical examination.
3. Formulate a differential diagnosis for pelvic organ prolapse, stress urinary incontinence, and mixed urinary incontinence.
4. Describe the various treatment options for the following pelvic floor disorders: pelvic organ prolapse, stress urinary incontinence, and mixed urinary incontinence.

Citation:
Kikuchi JY, Le Neveu M, Arnold S, et al. Creation and implementation of virtual urogynecology patient cases for medical student education. *MedEdPORTAL*. 2022;18:11259.
https://doi.org/10.15766/mep_2374-8265.11259

Introduction

Pelvic floor disorders, such as pelvic organ prolapse and urinary incontinence, affect 45% of women 30-90 years of age.[1] These disorders have a significant economic impact. In the United States from 2005 to 2006, the ambulatory cost of pelvic floor disorders was nearly $300 million,[2] and the impact of prolapse is expected to increase as the population ages.[3] Physicians specializing in urology and urogynecology treat a minority of women with various pelvic floor disorders, and practitioners in various medical and surgical specialties care for women with pelvic floor disorders.[4] Thus, it is important for all medical students to have a basic understanding of pelvic organ prolapse and urinary incontinence.

Virtual medical education has been studied extensively over the past decade and has become especially relevant during the COVID-19 pandemic, which has caused widespread

Copyright © 2022 Kikuchi et al. This is an open-access publication distributed under the terms of the Creative Commons Attribution license.

changes throughout all levels of medical education.[5] To provide students with the opportunity to practice their clinical skills in a controlled environment, many medical schools have incorporated virtual patient simulations into their existing curricula; however, within the field of urogynecology, the literature examining the use and efficacy of virtual training for medical students is very limited. There is currently only one study examining the learning outcomes of an online module for medical students in urogynecology.[6] This study created and validated an electronic learning module on urinary incontinence, which reviews female genitourinary embryologic development, anatomy, physiology, diagnosis, and management. It does not include virtual patient cases or address other pelvic floor disorders. Lastly, the electronic learning module is not publicly accessible.[6] Additionally, the Association of Professors of Gynecology and Obstetrics (APGO) have two educational videos on urinary incontinence and pelvic organ prolapse available for medical students; however, these videos are limited and do not utilize a case-based approach.[7,8] This paucity of resources demonstrates an important educational gap to address in medical student education. Lastly, medical education is notable for learning by opportunity, and clinical training alone cannot guarantee students' exposure to specific medical conditions during their in-person clerkships. Patient acceptance of medical student involvement in obstetrics and gynecology can further limit learning opportunities. One study of women presenting for gynecology appointments revealed that 26% are not willing to be seen by a medical student and 63% do not wish to have a gynecology exam performed by a medical student.[9] If medical students do not encounter patients with pelvic organ prolapse or various types of incontinence during their obstetrics and gynecology rotation, they may never learn to identify or address these prevalent women's health issues.

We created and incorporated virtual patient cases into the obstetrics and gynecology clinical clerkship to overcome this opportunity gap in urogynecologic clinical education. This interactive resource is a unique contribution to the literature. Upon searching PubMed, Google Scholar, and APGO, we found no other publicly accessible modules using a case-based approach for learning the evaluation and management of pelvic floor disorders.

Methods

During the spring of 2020, we created three virtual and interactive urogynecologic patient cases to augment in-person learning for all medical students participating in the obstetrics and gynecology clerkship at the Johns Hopkins University School of Medicine and the Penn State College of Medicine. These cases were created as teaching and assessment activities with complexity tailored to the learners' education level. The following medical diagnoses were covered in at least one of the three virtual patient cases: pelvic organ prolapse, stress urinary incontinence, and mixed urinary incontinence. A unique aspect of these virtual cases was that clinical reasoning was emphasized at each step of the case. Specifically, students were frequently prompted to reflect on patient health information, physical examination findings, and additional laboratory or radiologic workups. The students were asked to generate specific questions throughout the history and physical, to maintain appropriate communication with the virtual patients, and to formulate a differential diagnosis and treatment plan. We then integrated these virtual patient cases into the obstetrics and gynecology clerkship for the 2020-2021 academic year.

Goals of Virtual Patient Cases

We designed the virtual cases to help students develop skills in evaluating and managing patients with various pelvic floor disorders. Specifically, the cases taught medical students how to interview a patient, obtain an appropriate history, identify portions of a comprehensive urogynecologic examination, evaluate pelvic floor disorders, formulate a differential diagnosis, and describe various treatment options.

Creation of Virtual Patient Cases

Three interactive virtual patient cases were created by our team of urogynecology physicians from two different academic institutions: the Johns Hopkins University School of Medicine and the Penn State College of Medicine. When creating these cases, we specifically developed clinical scenarios routinely encountered in urogynecology outpatient clinics. Each case led the medical student through a urogynecology office visit. Throughout the case, students were asked to formulate questions, make note of pertinent information, review and record their findings, and devise an assessment and plan of care. The virtual patient cases and their pelvic floor disorders included the following:

- Mrs. Green: mixed urinary incontinence (Appendix A).
- Mrs. Smith: stress urinary incontinence (Appendix B).
- Mrs. Jones: anterior pelvic organ prolapse (Appendix C).

We provided instructions to view and access each interactive virtual patient case through a web browser (Appendices A-C). Each virtual patient case began with a chief complaint. The learner was prompted to write down questions they would ask to obtain a thorough history of present illness (HPI) and past

Copyright © 2022 Kikuchi et al. This is an open-access publication distributed under the terms of the Creative Commons Attribution license.

medical, surgical, obstetric, gynecologic, family, and social history. The learner was then guided through a physical exam with a specific focus on its gynecologic portion. Pictures of certain portions of the exam were provided. The learner was prompted on indications for any laboratory or radiologic workup, and estimates on the cost of these diagnostic tests were provided. Throughout the cases, the learner was stopped to give them time to reflect on the information presented and record any questions they might have. After creating a differential diagnosis, the learner was provided with explanations to guide them to the most likely diagnosis. The range of treatment options from conservative management to surgery were then presented. During this portion of the virtual patient cases, the learner was prompted to review the different treatment options with the virtual patient and ultimately determine which treatment option aligned best with the patient's needs, lifestyle, and health goals. Finally, at the conclusion of the exercise, a summary document was provided to emphasize learning objectives and summarize the entire case.

Utilization of the Interactive Online Platform

We developed the virtual patient cases in the Articulate Rise 360 course e-learning platform (Articulate Global Incorporated) used by the Johns Hopkins Office of Online Education.[10] This platform allowed for efficient and effective development of responsive case scenarios.

During case development in Rise 360, we first created pages that included pertinent information for each major section of the cases, directional language as appropriate, and/or clinical reasoning prompts. We developed these elements using text- and image-based, as well as interactive, elements, such as accordions and buttons. Continue button dividers were added between various elements on most pages to assist with controlling the flow of the learner's navigation through the content. In many instances, this required the learner to review all the content above the Continue button before proceeding to the next section.

After each case was developed in the Rise 360 platform, a case summary document with all the patient information was created and embedded as a media element in the Assessment page of each case. We distributed the cases via Rise 360 links.

Implementation of Virtual Patient Cases

When medical students were removed from all clinical rotations in the spring of 2020, we piloted these virtual cases among those students participating in a fully virtual urogynecology elective. We went on to integrate the cases into the obstetrics and gynecology clerkship for medical students during the 2020-2021 academic year. These modules were specifically utilized by students participating in the 2-week urogynecology rotation during this clerkship. Prior to their starting the urogynecology rotation, we provided the medical students with a guide that reviewed expectations and instructions for completing the virtual patient cases (Appendix D). The guide also included three articles for the students to read prior to completing the cases.[11-13] We instructed the medical students to independently complete the virtual patient cases during their urogynecology rotation. All three cases were available to the students starting at the beginning of the urogynecology clerkship and were accessible throughout the duration of the clerkship. The students were encouraged to complete the cases, but they did not receive any formal reminders regarding completion of the cases.

Debriefing Sessions With Faculty

Following completion of the virtual patient cases, the medical students met with one of the board-certified urogynecology faculty for debriefing sessions (Appendix E). These sessions were virtual, lasting approximately 45 minutes, and took place within 1 week of completing all three virtual patient cases. During these sessions, the students gave a comprehensive oral presentation of at least one virtual patient case. Afterwards, the faculty addressed their questions and ensured that the students met their learning objectives. We did not systematically collect data from the faculty debrief sessions. Retrospectively, faculty were interviewed about strengths and common gaps identified from the debriefing sessions with the students.

Evaluation Strategy

We developed a student survey through Qualtrics software version [May 2021] (Qualtrics) with a 5-point Likert scale (1 = *strongly disagree,* 5 = *strongly agree*) and open-ended questions to address the students' perceptions of (1) the usefulness of the cases, (2) the formatting of the cases, (3) their comfort level with pelvic floor disorders, and (4) overall feedback on the cases (Appendix F). We piloted the survey for clarity with medical students and faculty who did not participate in the curriculum. After completion of the obstetrics and gynecology clerkship, we emailed the survey link to all participants, and their responses were collected anonymously. The students were assured that the survey was voluntary and that responses were anonymous and would not impact their clerkship evaluations. We sent a second reminder email to complete the survey approximately 1 week after the initial email. The survey results were collected by a team member who was not involved in evaluating the students. This project was exempted by the local institutional review board.

Copyright © 2022 Kikuchi et al. This is an open-access publication distributed under the terms of the Creative Commons Attribution license.

Results

The urogynecology virtual patient cases were administered to all medical students participating in the urogynecology rotation of the obstetrics and gynecology clerkship between July 2020 and July 2021 and to one first-year medical student participating in a urogynecology elective. Our Qualtrics survey was administered to all 40 participating students, and 52% of students ($n = 21$) returned the questionnaire. There were two incomplete surveys.

The participants included a first-year medical student ($n = 1$), second-year medical students ($n = 3$), and third-year medical students ($n = 17$). A breakdown of participants' education level, experience with online learning courses, and interest in pursuing particular medical specialties is given in Table 1.

Table 1. Characteristics of Survey Participants ($n = 21$)

Characteristic	No. (%) of Participants
Level of education	
First-year medical student	1 (5)
Second-year medical student	3 (14)
Third-year medical student	17 (81)
Experience with online learning courses	
Before COVID-19	8 (38)
Since COVID-19	10 (48)
None	3 (14)
Interested in pursuing which medical specialty	
Dermatology	1 (5)
Emergency medicine	1 (5)
Family medicine	1 (5)
Internal medicine	4 (19)
OB/GYN	2 (10)
Pathology	1 (5)
Pediatrics	1 (5)
Radiology	1 (5)
Surgical-related specialty (general surgery, orthopedics, otolaryngology, ophthalmology, neurosurgery, urology, plastics, vascular, etc.)	6 (29)
Undecided	3 (14)

Quantitative Findings

Overall, the responses to the virtual patient cases were very positive. Ninety percent of the students surveyed ($n = 19$) agreed or strongly agreed that they were satisfied with the cases, and 71% ($n = 15$) agreed or strongly agreed that they would recommend the virtual patient cases to other students. All students ($n = 21$) felt that the format of the cases was easy to use and reported that the cases were appropriate for their level of learning. While the students had an overall positive response to the cases, only 24% ($n = 5$) felt that the cases could take the place of a urogynecology rotation. Table 2 presents the survey results regarding student perception of the usefulness of the cases.

Students were also surveyed on how the cases impacted their comfort level with their knowledge of common pelvic floor disorders and with educating patients about these disorders. In general, students felt that the virtual patient cases improved their confidence level with obtaining a patient's HPI, with the components of a urogynecologic physical exam, and with creating a differential diagnosis. Most students felt that the cases increased or significantly increased their confidence regarding nonsurgical management options for urinary incontinence ($n = 20$) and pelvic organ prolapse ($n = 20$). Fewer, though still a majority of students, reported improvement in their confidence levels for surgical management options for each of these disorders. The results for student comfort levels with pelvic floor disorders are displayed in Table 3.

Qualitative Findings

In the open-response section of the survey, students reported what they most enjoyed and disliked about the virtual patient cases, as well as how they would suggest improving them. The students' comments were categorized along similar themes, which are presented below. Select students' comments are also shown. For brevity, repetitive or similar comments have been omitted.

Table 2. Students' Perceptions of the Usefulness and Format of Cases ($n = 21$)

Survey Item	Strongly Disagree	Disagree	Neutral	Agree	Strongly Agree
Useful in supplementing my learning	0	1	3	4	12
Appropriate for my level of learning	0	0	0	6	14
Increased my interest in participating in an inpatient urogynecology rotation if offered in the future	0	5	7	3	4
Knowledge/skills gained will be useful in my future career	0	2	5	4	9
Can shorten the duration of an in-person urogynecology rotation	1	2	4	6	4
Can take the place of an in-person urogynecology rotation	5	8	2	1	4
Increased my interest in urogynecology and pelvic floor disorders	0	2	9	4	5
Would recommend these virtual patient cases to other students	0	0	5	8	7
Format was easy to use	0	0	0	9	12
Format was effective at accomplishing my learning goals	0	0	2	6	12
Appropriate for my level of learning	0	0	0	5	16
Overall satisfied with the virtual patient cases	0	0	1	9	10

Copyright © 2022 Kikuchi et al. This is an open-access publication distributed under the terms of the Creative Commons Attribution license.

Table 3. Students' Comfort Levels With Pelvic Floor Disorders ($n = 21$)

Survey Item	Topic Not Addressed in Cases Completed	Significantly Decreased Confidence	Decreased Confidence	No Change	Increased Confidence	Significantly Increased Confidence
Obtaining history of present illness	0	0	0	11	6	3
Urogynecology physical exam	0	0	0	3	12	5
Creating a differential diagnosis	0	0	0	1	10	9
Learning nonsurgical management options for urinary incontinence	0	0	0	0	10	10
Learning nonsurgical management options for pelvic organ prolapse	0	0	0	0	9	11
Learning surgical management options for urinary incontinence	1	0	0	1	10	8
Learning surgical management options for pelvic organ prolapse	0	0	0	1	10	9
Identifying risk factors associated with urinary incontinency and pelvic organ prolapse	0	0	0	2	11	7
Awareness of cost associated with laboratory and imaging workups commonly performed in urogynecology	0	0	0	3	8	9

In general, students found the cases to be a useful learning tool and liked that the cases could be completed asynchronously at the students' own pace:

- "In my experience, the cases were a fantastic learning tool for contextualizing the concepts, pathologies, and treatments that I needed to be familiar with to succeed on the rotation."
- "I enjoyed that they provided a systematic approach for different patient presentations and allowed me to work through the differential and management in my head before learning more."
- "Accessible any time. Allows use of additional resources simultaneously."

Students found the cases to be a valuable adjunct to, but not a substitute for, in-person care:

- "The virtual cases were useful to complete prior to the in-person rotation to have a better understanding of what I was seeing in the clinic and OR."
- "Helpful for seeing things I did not get much exposure to on my 3rd year OBGYN rotation."
- "While there is no true substitute to in person rotations, the virtual case format was as close of a substitute as I have had the opportunity to work with."
- "I don't think these would be a good alternative to in-person rotations because you miss out on interacting with the patients and seeing the procedures.... You also don't get to improve physical exam skills in the online format."

Students found the summary document provided after each case to be useful as a reference to review during and beyond their rotation:

- "The virtual cases gave me a good idea of what to expect in a urogyn clinic, including important questions to ask in the HPI, risk factors, differential diagnoses, work-up, and treatment options."
- "I appreciated the thoroughness of the online cases, and I also loved the summary document provided at the end of each case, which I could continue to review and reference during the rotation (and beyond)."

Students also identified areas for improvement. Regarding the format, some felt that there was too much time spent clicking through the pages, with some difficulty navigating back to previous sections; that some cases were more time consuming than others; and that some pages could be condensed to one page:

- "User format could be improved to easier allow you to click through and back the cases."
- "It took awhile to click through all the parts of the cases and perhaps some parts could be condensed on the same pages."

Students also felt that there could have been more explanation of diagnostic and treatment pathways and how to narrow the differential diagnosis:

- "Including more explanation for what the diagnostic pathway is, and the treatment pathways/alternatives are."
- "More pictures for physical exam components."

Urogynecology faculty were interviewed regarding their recollections of the strengths and common gaps identified during their debriefing sessions with students. Their responses were overwhelmingly positive. The faculty agreed that the

Copyright © 2022 Kikuchi et al. This is an open-access publication distributed under the terms of the Creative Commons Attribution license.

students' oral case presentations were well prepared and conveyed relevant clinical data. All the students expressed a basic understanding of pelvic organ prolapse and urinary incontinence. The most common gaps identified were physical exam findings. Students had the most questions regarding the logistics of performing and interpreting a urogynecologic pelvic exam, and several students verbalized a desire to solidify their learning with an in-person experience in the urogynecology clinic.

Discussion

We successfully developed a virtual learning activity in which learners can practice their skills caring for women with pelvic floor disorders. Our findings suggest that the virtual patient cases are an acceptable, valuable, and effective tool for learners. The interactive modules allow for self-paced practice, and our positive learner feedback indicates that the modules are accessible, beneficial for learning, and comprehensive. Although virtual cases cannot replace in-person patient interactions, utilizing these cases can help address the existing gap in exposure to pelvic floor disorders both due to limited access to subspecialty care and variability in exposure secondary to nature of learning by opportunity. Furthermore, widespread accessibility to these cases may serve to help mitigate international disparities in exposure to urogynecologic disorders that precedes the underrepresentation highlighted by the COVID-19 pandemic.

The feedback that we received from medical students was overwhelmingly positive, and rates of satisfaction reported among our students (90%) were similar to those described in other studies utilizing a virtual format.[14,15] Students applauded the accessibility and flexibility of the virtual patient cases, similar to the advantages of virtual learning that have been previously described in the literature.[16,17] Students found that the cases were clear, easy to use, and appropriate for their level of learning. In addition to utilizing an online platform to complete cases asynchronously at one's own pace, the virtual patient cases provide preemptive exposure to specific and separate urogynecology issues for learners, meeting the goal of increasing the knowledge and assessment of pelvic floor disorders. For potential improvements, we received student feedback regarding the navigability of the virtual patient cases, particularly with clicking through the cases. For future revisions, certain portions of the virtual cases, such as the HPI and physical exam, can be condensed to minimize the amount of clicking required. Additionally, it would be beneficial to expand the differential diagnosis portion to provide explanations as to why certain diagnoses are more or less likely. Lastly, the Guide for the Virtual Patient Cases (Appendix E) could be revised to incorporate the APGO video modules on pelvic organ prolapse and urinary incontinence[7,8] as additional learning resources, which can be valuable for those students who prefer video presentations for their learning. To minimize the amount of prereading material, the APGO video modules could be included as the required material and the American College of Obstetricians and Gynecologists Practice Bulletins[11,12] listed as optional reading assignments, since the bulletins are specifically designed for practicing physicians.

There are several limitations to the use of these virtual patient cases. Most notably, our survey had a low response rate of 52%. The survey was administered after completion of the obstetrics and gynecology clerkship, and students were not required to complete it; therefore, results gathered are subject to response bias. Additionally, our evaluation was perceptions focused, and we did not utilize a standardized method to assess learning. Future iterations should incorporate a knowledge-based assessment, such as a series of questions testing knowledge of pelvic organ prolapse and urinary incontinence in accordance with APGO learning objectives. We also did not systematically collect data from the faculty debrief sessions, which could have provided robust data. Retrospectively, faculty were interviewed about the strengths and common gaps identified. Although not explicitly mentioned in the students' written feedback, completing the cases also requires reliable access to the Internet which may be a barrier for some learners. Concerns have been raised regarding reduced student engagement and focus when using virtual teaching platforms,[18] a sentiment that was reiterated in some students' responses. Time to complete the cases was not assessed, which could potentially guide further improvements in the case format. Furthermore, because participation was neither tracked nor mandated, variable levels of engagement with the cases may impact conclusions drawn from the survey responses.

Most students felt that the cases improved their confidence regarding nonsurgical management options for pelvic floor disorders. Fewer reported improvement in confidence regarding surgical management options for each of these disorders. Historically, technical difficulties encountered during virtual learning have been a significant limitation.[18,19] While students who completed our virtual patient cases did not describe technical difficulties, they did identify opportunities to improve usability by adjusting formatting to improve navigation through the cases that were incorporated into the final version. To optimize this learning tool, debriefing sessions with faculty were added to address feedback regarding comparisons across various pelvic floor disorders and navigating diagnostic pathways.

Copyright © 2022 Kikuchi et al. This is an open-access publication distributed under the terms of the Creative Commons Attribution license.

The online nature of the platform precludes students from practicing a physical exam, participating in procedures, and gleaning from bedside clinical teaching. Overcoming this barrier is essential for comprehensive student learning, and future efforts spent developing strategies to combat this challenge would be beneficial. While we were able to obtain feedback from students after they completed their clerkship, the long-term impact of virtual learning on educational gaps of medical students remains unknown.

Conclusion

Innovation in the era of the COVID-19 pandemic has challenged educators to develop virtual online learning opportunities to augment in-person clinical learning. While the urogynecology modules effectively mitigate this educational gap, we assert that the exposure to multiple gynecologic disease states supersedes the learning challenges introduced with the COVID-19 pandemic and can be used to effectively combat existing educational inequities in exposure to these important yet often overlooked women's health topics in the medical student curriculum.

Appendices

A. Case 1 Mixed Urinary Incontinence folder
B. Case 2 Stress Urinary Incontinence folder
C. Case 3 Pelvic Organ Prolapse folder
D. Guide for Virtual Patient Cases.docx
E. Guide for Faculty Debriefing Session.docx
F. Survey for Virtual Cases.docx

All appendices are peer reviewed as integral parts of the Original Publication.

Jacqueline Y. Kikuchi, MD: Fellow, Department of Gynecology and Obstetrics, Johns Hopkins University School of Medicine; ORCID: https://orcid.org/0000-0003-2357-4675

Margot Le Neveu, MD: Third-Year Resident, Department of Gynecology and Obstetrics, Johns Hopkins University School of Medicine

Shannon Arnold, MSc: Student, Post-Baccalaureate Premedical Program, Johns Hopkins University

Austin Offnick: Student, Post-Baccalaureate Premedical Program, Johns Hopkins University

Keila S. Muñiz, MD: Fellow, Department of Gynecology and Obstetrics, Johns Hopkins University School of Medicine

Prerna Pandya, MD: Fellow, Department of Gynecology and Obstetrics, Johns Hopkins University School of Medicine

Rehan Feroz, DO: Third-Year Resident, Department of Obstetrics and Gynecology, Penn State College of Medicine

Jaime B. Long, MD: Assistant Professor, Department of Obstetrics and Gynecology, Penn State College of Medicine

Lindsay R. Ledebur, MA: Instructional Designer, Office of Online Education, Johns Hopkins University School of Medicine

Danielle Patterson, MD: Assistant Professor, Department of Gynecology and Obstetrics, Johns Hopkins University School of Medicine

Chi Chiung Grace Chen, MD, MHS: Associate Professor, Department of Gynecology and Obstetrics, Johns Hopkins University School of Medicine

Disclosures
None to report.

Funding/Support
None to report.

Ethical Approval
The Johns Hopkins Medicine Institutional Review Board deemed further review of this project not necessary.

References

1. Melville JL, Delaney K, Newton K, Katon W. Incontinence severity and major depression in incontinent women. *Obstet Gynecol*. 2005;106(3):585-592. https://doi.org/10.1097/01.AOG.0000173985.39533.37
2. Sung VW, Washington B, Raker CA. Costs of ambulatory care related to female pelvic floor disorders in the United States. *Am J Obstet Gynecol*. 2010;202(5):483.E1-483.E4. https://doi.org/10.1016/j.ajog.2010.01.015
3. Wu JM, Hundley AF, Fulton RG, Myers ER. Forecasting the prevalence of pelvic floor disorders in U.S. women: 2010 to 2050. *Obstet Gynecol*. 2009;114(6):1278-1283. https://doi.org/10.1097/AOG.0b013e3181c2ce96
4. Mueller ER, Kenton K, Rogers RG, Fenner DE. Are we missing an opportunity to teach future physicians about female pelvic floor disorders? *Int Urogynecol J Pelvic Floor Dysfunct*. 2009;20(12):1413-1415. https://doi.org/10.1007/s00192-009-0966-y
5. Akers A, Blough C, Iyer MS. COVID-19 implications on clinical clerkships and the residency application process for medical students. *Cureus*. 2020;12(4):e7800. https://doi.org/10.7759/cureus.7800
6. Parker-Autry CY, Shen E, Nance A, et al. Validation and testing of an e-learning module teaching core urinary incontinence objectives in a randomized controlled trial. *Female Pelvic Med Reconstr Surg*. 2019;25(2):188-192. https://doi.org/10.1097/SPV.0000000000000695

Copyright © 2022 Kikuchi et al. This is an open-access publication distributed under the terms of the Creative Commons Attribution license.

7. Association of Professors of Gynecology and Obstetrics (APGO). APGO basic sciences - topic 24: pelvic organ prolapse. YouTube. 2018. https://youtu.be/30TiM72_8GA
8. Association of Professors of Gynecology and Obstetrics (APGO). APGO basic sciences - topic 29: urinary incontinence. YouTube. 2018. https://youtu.be/YgYtgnKhK9I
9. Fortier AM, Hahn PM, Trueman J, Reid RL. The acceptance of medical students by women with gynaecology appointments. *J Obstet Gynaecol Can*. 2006;28(6):526-530. https://doi.org/10.1016/S1701-2163(16)32179-X
10. Rise 360. Articulate 360. Accessed 2020. https://articulate.com/360/rise
11. Practice Bulletin no. 155: urinary incontinence in women. *Obstet Gynecol*. 2015;126(5):e66-e81. https://doi.org/10.1097/AOG.0000000000001148
12. Pelvic organ prolapse: ACOG Practice Bulletin no. 214. *Obstet Gynecol*. 2019;134(5):e126-e142. https://doi.org/10.1097/AOG.0000000000003519
13. Committee Opinion no. 603: evaluation of uncomplicated stress urinary incontinence in women before surgical treatment. *Obstet Gynecol*. 2014;123(6):1403-1407. https://doi.org/10.1097/01.AOG.0000450759.34453.31
14. Sud R, Sharma P, Budhwar V, Khanduja S. Undergraduate ophthalmology teaching in COVID-19 times: students' perspective and feedback. *Indian J Ophthalmol*. 2020;68(7):1490-1491. https://doi.org/10.4103/ijo.IJO_1689_20
15. Kaur N, Dwivedi D, Arora J, Gandhi A. Study of the effectiveness of e-learning to conventional teaching in medical undergraduates amid COVID-19 pandemic. *Natl J Physiol Pharm Pharmacol*. 2020;10(7):563-567. https://doi.org/10.5455/njppp.2020.10.04096202028042020
16. Dedeilia A, Sotiropoulos MG, Hanrahan JG, Janga D, Dedeilias P, Sideris M. Medical and surgical education challenges and innovations in the COVID-19 era: a systematic review. *In Vivo*. 2020;34(3 suppl):1603-1611. https://doi.org/10.21873/invivo.11950
17. Sahi PK, Mishra D, Singh T. Medical education amid the COVID-19 pandemic. *Indian Pediatr*. 2020;57(7):652-657. https://doi.org/10.1007/s13312-020-1894-7
18. Wilcha RJ. Effectiveness of virtual medical teaching during the COVID-19 crisis: systematic review. *JMIR Med Educ*. 2020;6(2):e20963. https://doi.org/10.2196/20963
19. Johnson CM, Corazzini KN, Shaw R. Assessing the feasibility of using virtual environments in distance education. *Knowl Manag Elearn*. 2011;3(1):5-16. https://doi.org/10.34105/j.kmel.2011.03.002

Received: December 3, 2021
Accepted: April 7, 2022
Published: May 27, 2022

Copyright © 2022 Kikuchi et al. This is an open-access publication distributed under the terms of the Creative Commons Attribution license.
