# Supplementary material for: Creation and Implementation of Virtual Urogynecology Patient Cases for Medical Student Education
Source: MedEdPORTAL. 2022 May 27;18:11259. doi: 10.15766/mep_2374-8265.11259 (PMC9135914; doi:10.15766/mep_2374-8265.11259)
Supplement: Supplementary file 1 — Case 1 Mixed Urinary Incontinence folderCase 2 Stress Urinary Incontinence folderCase 3 Pelvic Organ Prolapse folderGuide for Virtual Patient Cases.docxGuide for Faculty Debriefing Session.docxSurvey for Virtual Cases.docx [file mep_2374-8265.11259-s001.zip › F. Survey for Virtual Cases.docx]

**Survey for Virtual Cases**

**Demographic Questions:**

1. What gender do you identify as?

- Female
- Male
- Nonbinary / third gender
- Prefer not to say

1. What is your age?

- _________

1. Have you had experience with any other exclusively online learning courses before completing the virtual cases?

- Yes, prior to COVID-19
- Yes, since COVID-19
- No
- Other (please specify): _______________

1. In which year of medical school did you complete the virtual patient cases?

- Pre-med
- 1st year
- 2nd year
- 3rd year
- 4th year
- Other (please specify): __________________

1. What medical specialty are you planning to pursue?

- Anesthesiology
- Dermatology
- Emergency Medicine
- Family Medicine
- Internal Medicine
- Neurology
- Ob/Gyn
- Pathology
- Pediatrics
- Psychiatry
- Radiation Oncology
- Radiology
- Surgical-related specialty (General Surgery, Orthopedics, Otolaryngology, Ophthalmology, Neurosurgery, Urology, Plastics, Vascular, etc).
- Undecided
- Other (please specify): _______________________

1. Which medical rotations have you completed (prior to completing the virtual cases)? (select all that apply)

- Pre-med only
- Pre-clinical work only
- Anesthesiology
- Dermatology
- Emergency Medicine
- Family Medicine
- Internal Medicine
- Neurology
- Ob/Gyn
- Pathology
- Pediatrics
- Psychiatry
- Radiation Oncology
- Radiology
- Surgical-related specialty (General Surgery, Orthopedics, Otolaryngology, Ophthalmology, Neurosurgery, Urology, Plastics, Vascular, etc).
- Other (please specify): _______

**Case-Related Questions:**

Please evaluate the following statements in regard to your experience with the virtual cases.

1. The format of the virtual cases was easy to use.


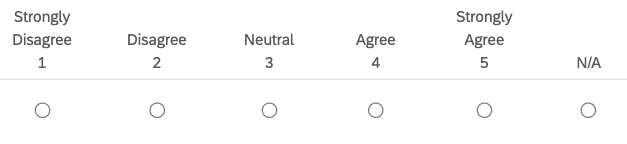


1. The format of the virtual cases was effective at accomplishing my learning goals.


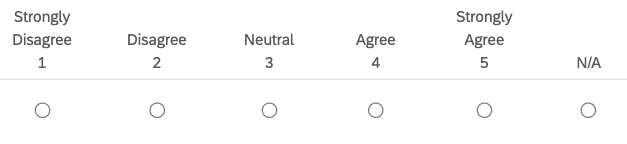


1. The virtual cases were appropriate for my level of learning.


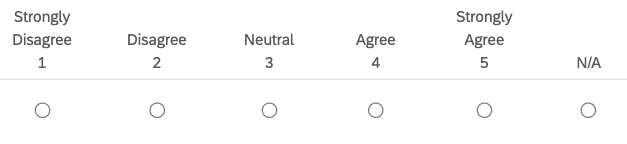


1. The additional case resources were useful in supplementing my learning.


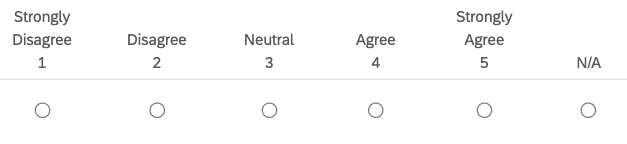


1. The synchronous virtual debriefing which occurred after reviewing the cases was an essential aspect of my learning.


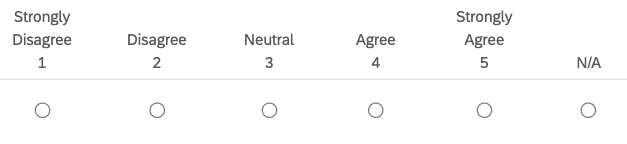


1. The knowledge/skills that I gained from the virtual cases will be useful in my future career.


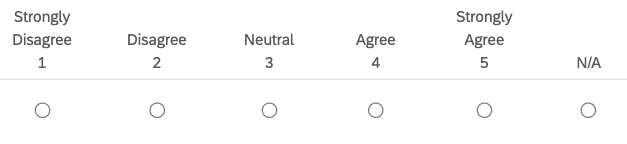


1. The virtual cases were useful in advancing my knowledge of urogynecology and pelvic floor disorders.


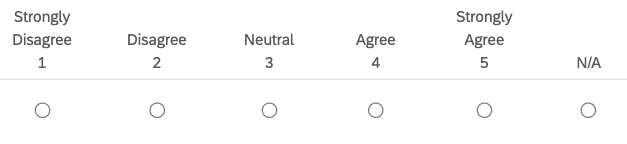


1. The virtual cases increased my interest in urogynecology and pelvic floor disorders.


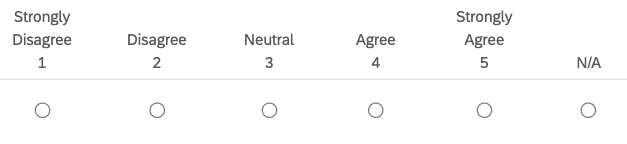


1. The virtual cases increased my interest in participating in an in-person urogynecology rotation if offered in the future.


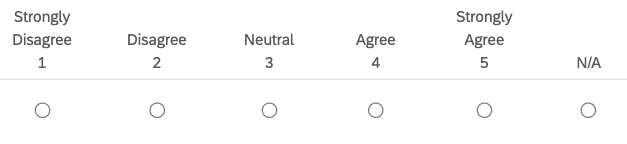


1. The virtual cases can take the place of an in-person urogynecology rotation.


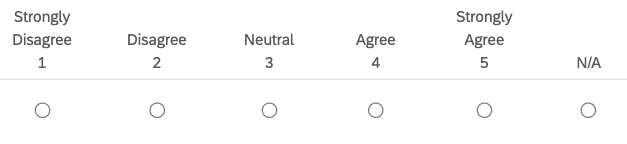


1. The virtual cases can shorten the duration of an in-person urogynecology rotation.


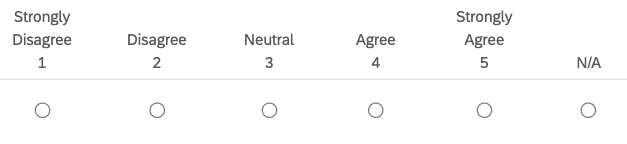


1. I would recommend the virtual cases to other students.


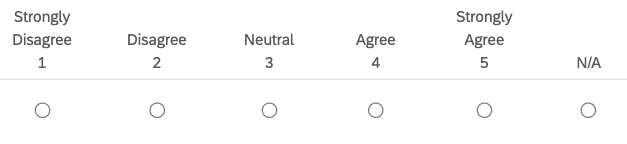


1. As an overall impression, I was satisfied with the virtual cases.


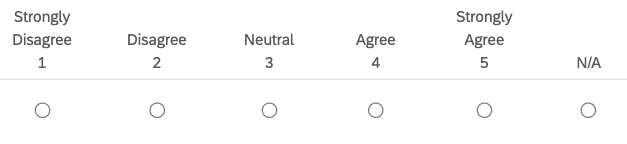


**Self-Assessment Questions:**

Compared with your own baseline comfort level with each of the following topics, how did completing the virtual cases affect your progress towards the following learning objectives?

1. Obtaining a patient’s HPI


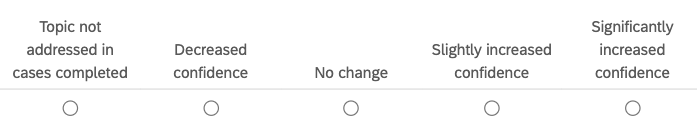


1. Components of gynecologic and urogynecologic physical exam


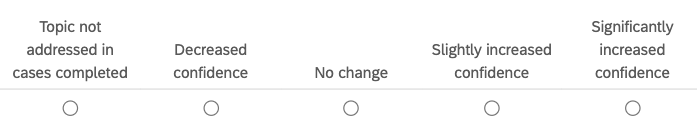


1. Creating a differential diagnosis


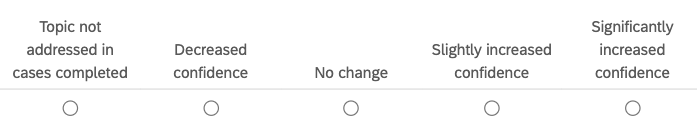


1. Non-surgical management options for urinary incontinence


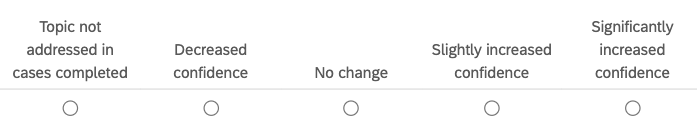


1. Non-surgical management options for pelvic organ prolapse


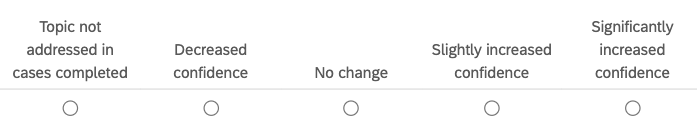


1. Surgical management options for urinary incontinence


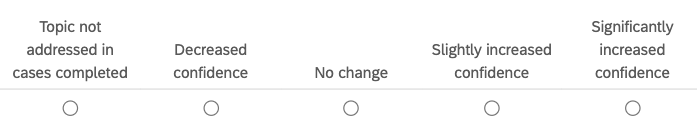


1. Surgical management options for pelvic organ prolapse


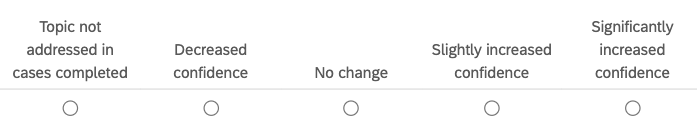


1. Identifying risk factors associated with urinary incontinence and pelvic organ prolapse


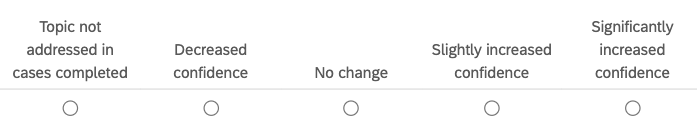


1. Awareness of cost associated with laboratory and imaging work-ups commonly performed in urogynecology


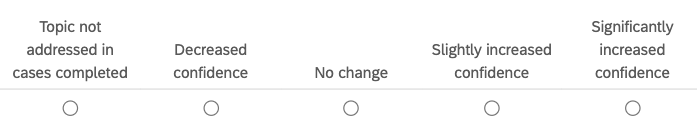


**Open-Ended Questions:**

1. What did you enjoy about the virtual cases?
2. What did you dislike about the virtual cases?
3. What did you find to be the pros and cons of the virtual case format vs. an in-person rotation? Please elaborate if applicable.
4. Do you have any suggestions for improving the virtual cases?
