# Supplementary figures and images for: Creation and Implementation of Virtual Urogynecology Patient Cases for Medical Student Education
Source: MedEdPORTAL. 2022 May 27;18:11259. doi: 10.15766/mep_2374-8265.11259 (PMC9135914; doi:10.15766/mep_2374-8265.11259)

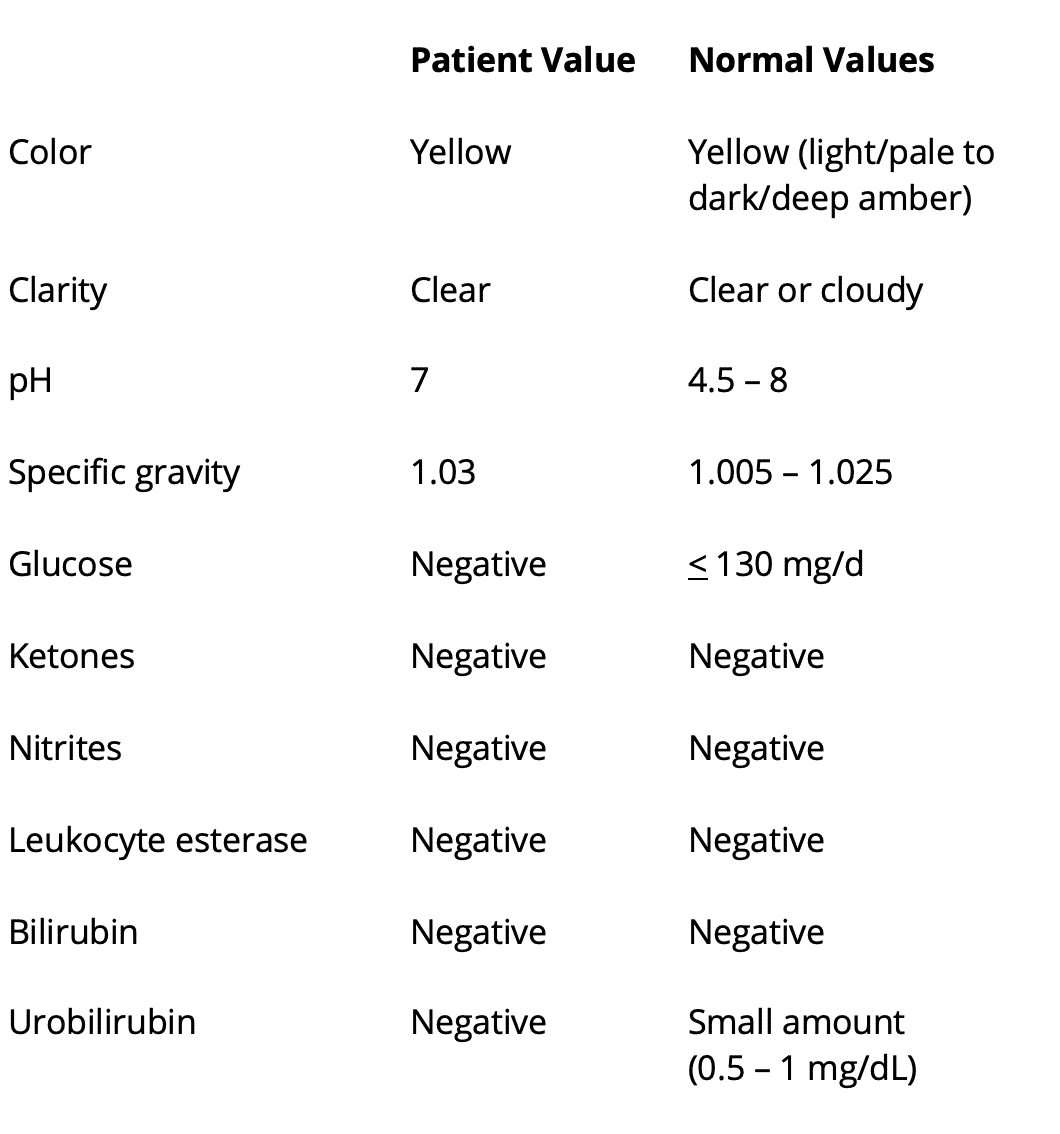

Supplement: Supplementary file 1 — Case 1 Mixed Urinary Incontinence folderCase 2 Stress Urinary Incontinence folderCase 3 Pelvic Organ Prolapse folderGuide for Virtual Patient Cases.docxGuide for Faculty Debriefing Session.docxSurvey for Virtual Cases.docx [file mep_2374-8265.11259-s001.zip › A. Case 1 Mixed Urinary Incontinence/content/assets/bvchfSmjpRIUeKXl_17jmZIwNEoxz_J8_.png]

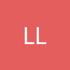

Supplement: Supplementary file 1 — Case 1 Mixed Urinary Incontinence folderCase 2 Stress Urinary Incontinence folderCase 3 Pelvic Organ Prolapse folderGuide for Virtual Patient Cases.docxGuide for Faculty Debriefing Session.docxSurvey for Virtual Cases.docx [file mep_2374-8265.11259-s001.zip › A. Case 1 Mixed Urinary Incontinence/content/assets/CikUYNEhka8r0T9B_small.png]

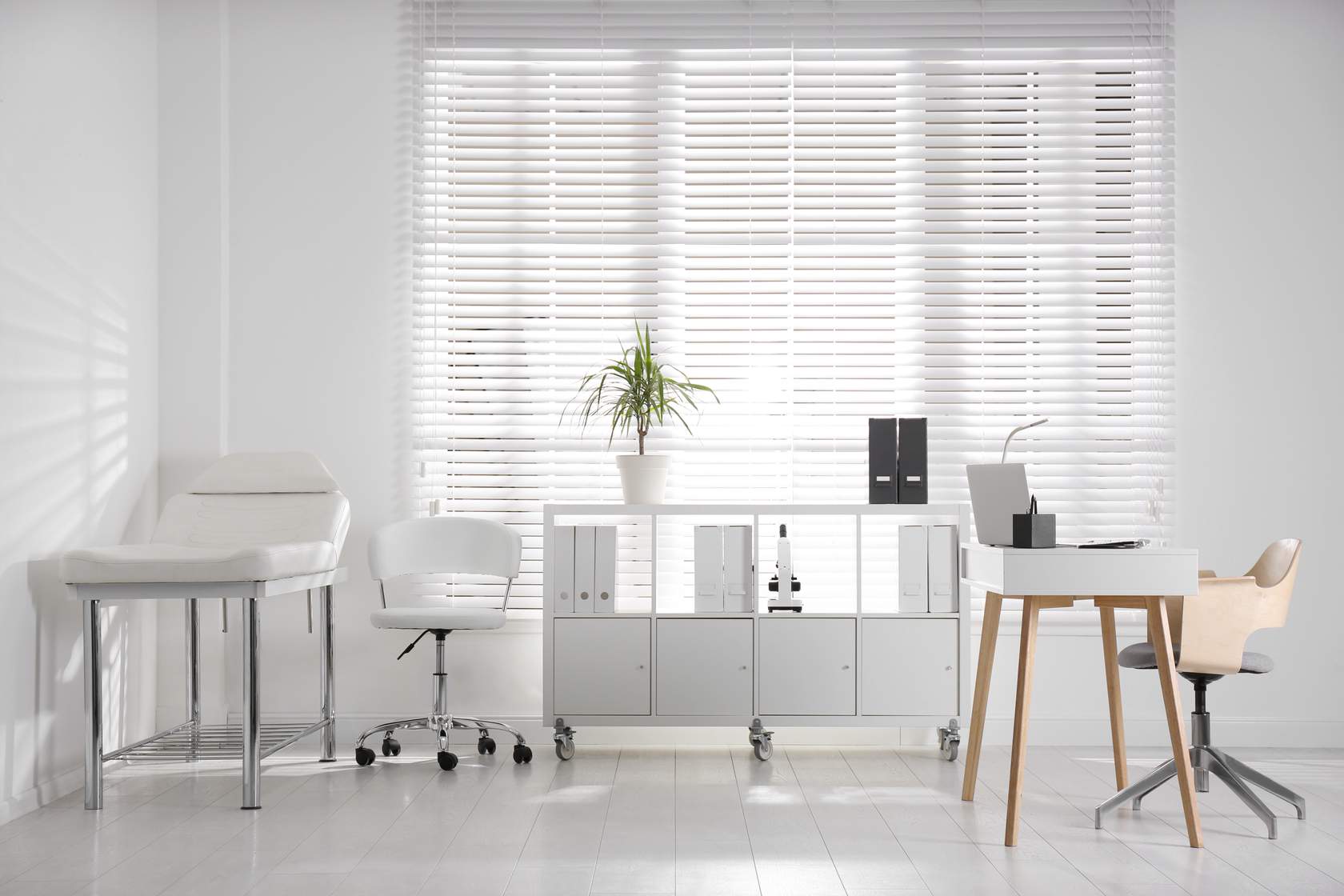

Supplement: Supplementary file 1 — Case 1 Mixed Urinary Incontinence folderCase 2 Stress Urinary Incontinence folderCase 3 Pelvic Organ Prolapse folderGuide for Virtual Patient Cases.docxGuide for Faculty Debriefing Session.docxSurvey for Virtual Cases.docx [file mep_2374-8265.11259-s001.zip › A. Case 1 Mixed Urinary Incontinence/content/assets/CLxddnq2Hv127xa-_e6xzlYDBO76yMIrh.jpg]

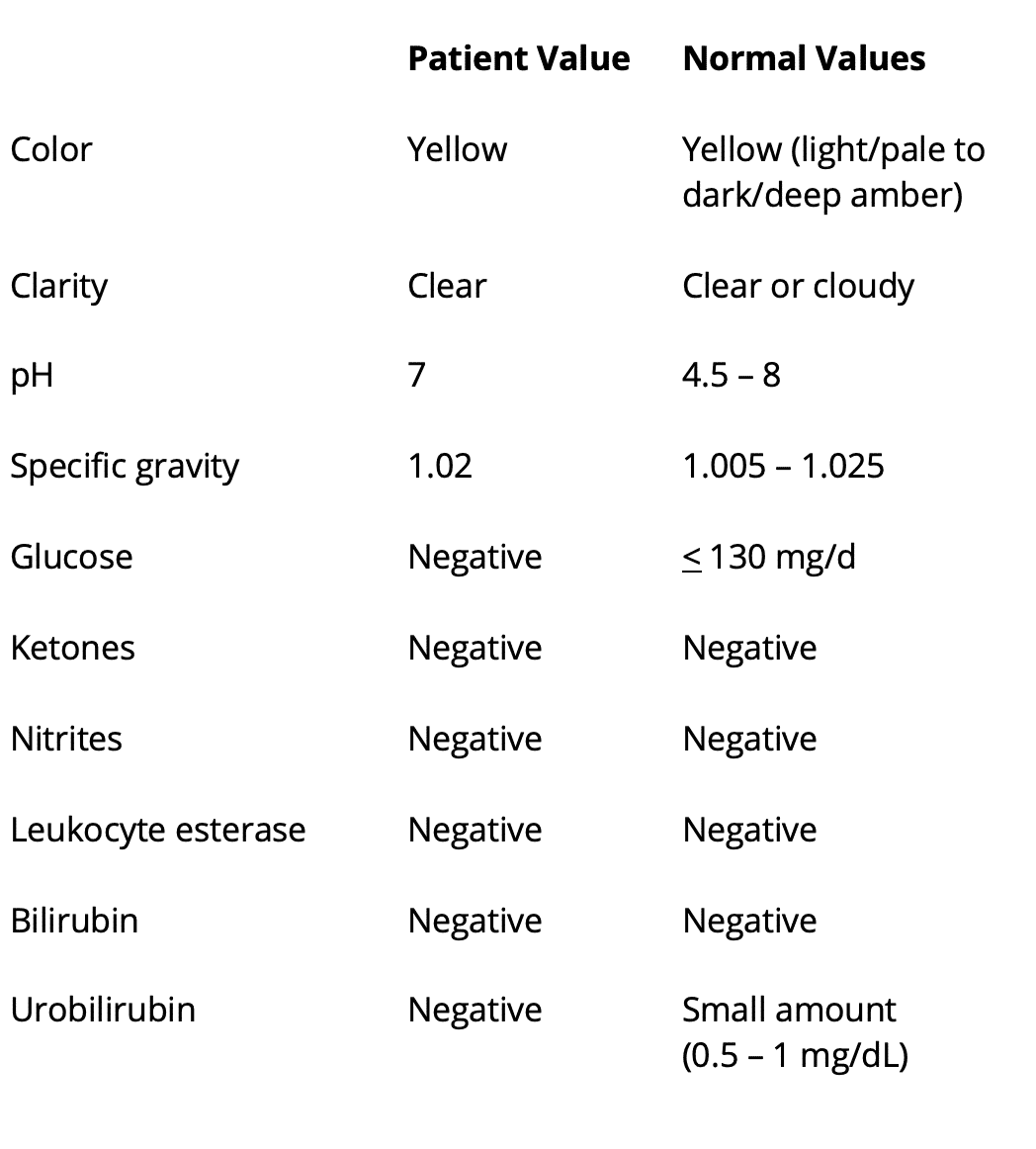

Supplement: Supplementary file 1 — Case 1 Mixed Urinary Incontinence folderCase 2 Stress Urinary Incontinence folderCase 3 Pelvic Organ Prolapse folderGuide for Virtual Patient Cases.docxGuide for Faculty Debriefing Session.docxSurvey for Virtual Cases.docx [file mep_2374-8265.11259-s001.zip › B. Case 2 Stress Urinary Incontinence/content/assets/6_pDoolgFe09PrR1_4fH2vDNT6apW0iV8.png]
